# Human Trefoil Factor 3 induces the transcription of its own promoter through STAT3

**DOI:** 10.1038/srep30421

**Published:** 2016-07-25

**Authors:** Yong Sun, Liangxi Wang, Yifang Zhou, Xuefei Mao, Xiangdong Deng

**Affiliations:** 1 Department of Burn Surgery, Huaihai Hospital affiliated to Xuzhou Medical College, Xuzhou 221004, China; 2 Department of Burn Surgery, No. 97 Hospital of PLA, Xuzhou 221004, China

## Abstract

Human trefoil factor 3 (hTFF3) is a small peptide of potential therapeutic value. The mechanisms underlying the transcriptional regulation of hTFF3 remain unclear. The purpose of this study was to identify the core functional elements for the self-induction action of hTFF3 and transcription factors. First, truncated promoters were constructed to identify the functional regions of the hTFF3 promoter. Next, point mutation, chromatin immunoprecipitation, RNA interference, and gene overexpression experiments were performed to analyze the transcriptional binding sites responsible for the self-induced transcription of hTFF3. Our results revealed the −1450 bp to −1400 bp fragment of the hTFF3 promoter was the functional region for the self-induction action of hTFF3. Bioinformatics analysis confirmed that a STAT3 binding site is present in the −1417 bp to −1409 bp region. Subsequently, site-directed mutagenesis analysis determined that this STAT3 binding site was critical for the self-induction effect of hTFF3. ChIP experiments confirmed that STAT3 binds to the hTFF3 promoter. STAT3 overexpression and knockdown experiments revealed that STAT3 enhanced the self-induction effect and the expression of hTFF3. This study confirmed that hTFF3 exhibits self-induction action, and that STAT3 is the key transcription factor to maintain the function of self-induction.

Human trefoil factor 3 (hTFF3) is a small polypeptide secreted by intestinal goblet cells. There are six cysteine residues in the amino acid sequence of hTFF3, sequentially connected in pairs by disulfide bonds to form three ring structures^1^. This structure confers stability, and thus provides resistance against acidic and basic conditions, as well as protease hydrolysis. The structural stability protects hTFF3 from damages in the gastrointestinal tract, which is a complex environment. hTFF3 has been under intensive research by numerous scholars since its discovery. A large number of studies have shown that hTFF3 plays imperative roles in the maintenance and repair of the intestinal mucosa^2^,^3^. The regulation of hTFF3 is complex and precise, and many kinds of substances are involved in the regulation of hTFF3 expression. Some studies have found that proteins of the trefoil factor family exhibit the phenomenon of “self-induction” to enhance their own expression^4^. However, the exact regulatory mechanisms remain unclear. The present study successfully amplified hTFF3 promoter fragments of varying lengths, and identified the −1450 to −1400 bp region as the functional region for its self-induction. Bioinformatics and site-directed mutagenesis analyses revealed that the STAT3 binding site, located in the region of −1417 to −1409 bp, is necessary for the self-induction of hTFF3. We further proved that STAT3 binds to hTFF3 promoter to regulate its transcription. Our study lays a foundation for elucidating the regulatory mechanisms of hTFF3.

## Results

### hTFF3 enhances the transcription of its promoter

**Figure 1:**
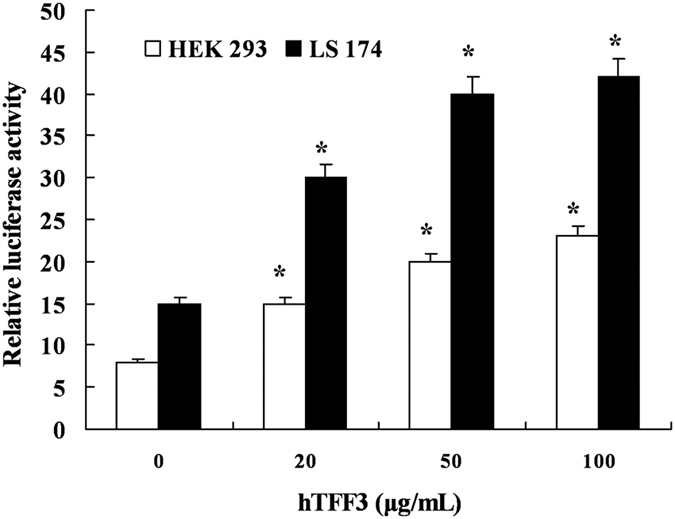
The self-induction effect of hTFF3. The full-length promoter of hTFF3 was transfected into HEK 293 cells and LS174T cells, and the transfected cells were stimulated with different concentrations of hTFF3 at 24 h post-transfection. The relative fluorescence intensity was calculated as the ratio of the firefly fluorescence intensity to *Renilla* fluorescence intensity. At least three independent experiments were performed under each condition for this experiment. Data are presented mean ± S.D. (*P < 0.05 compared with the control sample of 0 μg/mL).

Plasmids expressing the full-length hTFF3 promoter or control DNA were transfected into HEK293 cells and LS174T cells, and different concentrations of hTFF3 was added at 24 h post-transfection. The relative luciferase activity was measured using a dual-luciferase reporter system at 48 h. The results showed that the luciferase activity of cells expressing the hTFF3 promoter was 15-fold higher than that of the negative control expressing pGL3-basic, and the enhanced luciferase activity showed a dose-dependent effect with increasing concentrations of hTFF3 treatment. The luciferase activity of cells expressing the hTFF3 promoter was 45-fold higher than that of the negative control expressing pGL3-basic when treated with 50 μg/mL hTFF3. This difference was significant (P < 0.01). Moreover, the relative luciferase activity in LS174T cells was significantly higher than that in HEK293 cells (P < 0.01) (Fig. 1).

### Effects of hTFF3 on the transcription of its promoter fragments of varying lengths

**Figure 2:**
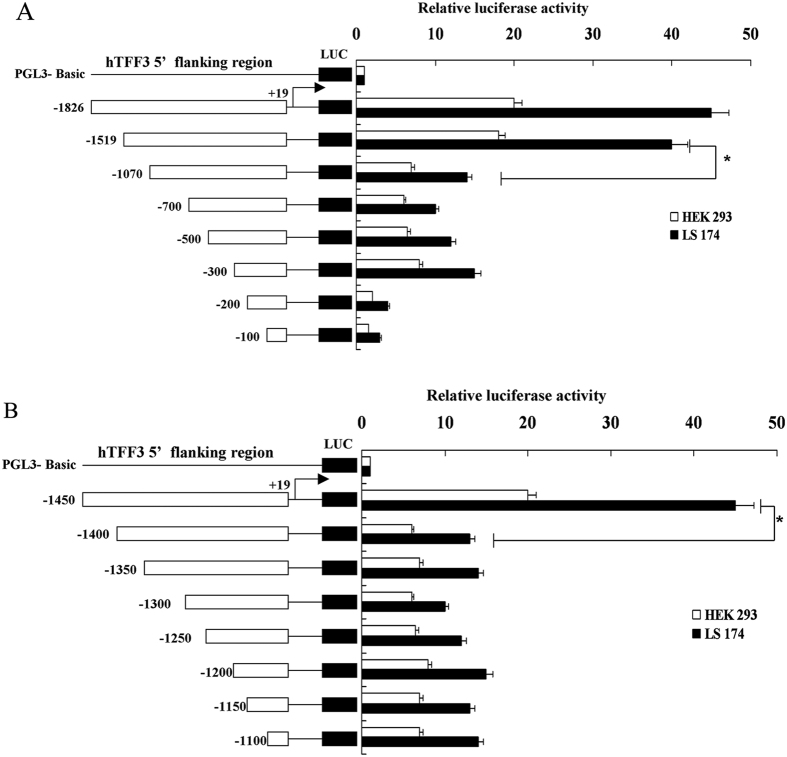
Effects of hTFF3 on the transcription of its promoter fragments of varying lengths. hTFF3 promoter fragments of varying lengths were transfected into HEK293 cells and LS174T cells, and the relative fluorescence intensity was calculated as the ratio of the firefly fluorescence intensity to *Renilla* fluorescence intensity. At least three independent experiments were performed under each condition. (**A**) Represents the promoter length of −1826 bp to −100 bp; (**B**) represents −1450 bp to −1100 bp. Data are presented mean ± S.D. (**A**) *P < 0.05 compared with pGL3−1070; (**B**) *P < 0.05 compared with pGL3−1400).

We transfected fragments of the hTFF3 promoter into LS174T 293 cells and HEK cells, and treated with exogenous hTFF3. It showed that the luciferase activity in LS174T cells was significantly higher than that in HEK293 cells, regardless of the length of the transfected promoter. The luciferase activities of cells expressing pGL3−1826 and pGL3−1519 were relatively higher, and the luciferase activities of cells expressing pGL3−1070 and shorter length fragments were significantly lower (Fig. 2A). Therefore, to further narrow down on the functional region, we constructed eight truncated fragments between −1519 bp and −1070 bp. As shown in Fig. 2B, cells expressing pGL3−1450 had a luciferase activity 41-fold more than that of cells expressing pGL3-basic, and cells expressing pGL3−1400 and GL3−1100 exhibited relatively low luciferase activities, which were only 10–15 fold higher than that of the cells expressing pGL3-basic.

### Mutations of −1417 bp to −1409 bp reduced transcription of the hTFF3 promoter

**Figure 3:**
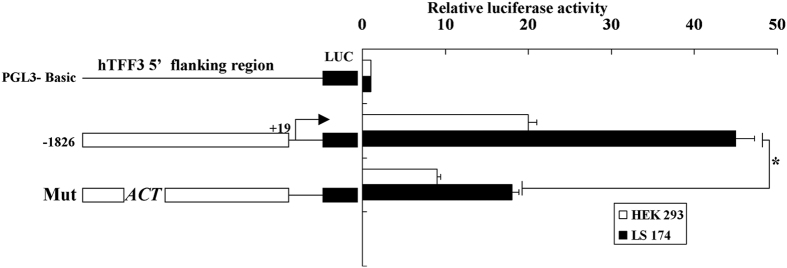
Mutation analysis of the hTFF3 promoter. The hTFF3 promoter was transfected into HEK293 cells and LS174T cells, and the relative fluorescence intensity was calculated as the ratio of the firefly fluorescence intensity to *Renilla* fluorescence intensity. Results were obtained from three independent experiments. Data are presented mean ± S.D. (*P < 0.05 compared with pGL3−1826).

We entered the DNA sequence of the −1450 to −1400 bp region of hTFF3 promoter into TFSEARCH database. Interestingly, a STAT3 binding site (TTCCTGGAA) was found in the region of −1417 bp to −1409 bp, with a score of 94.2. Therefore, we mutated the core region of the STAT3 binding site from CTG to ACT. As shown in Fig. 3, the luciferase activity of cells expressing pGL3−1826 was 45-fold higher than that of cells expressing pGL3-basic, while the activity of cells expressing the mutant reporter decreased to only 17-fold higher than that of cells expressing pGL3-basic.

### Verification of the binding activity of STAT3 to the hTFF3 promoter

**Figure 4:**
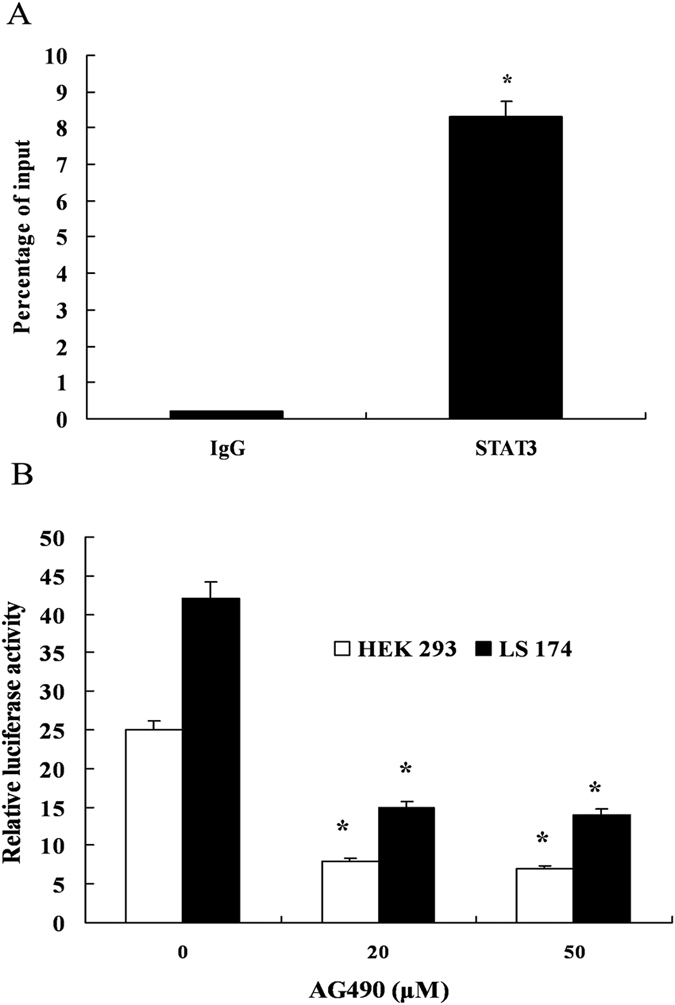
Verification of the binding activity of STAT3 to the hTFF3 promoter. (**A**) LS174Tcells were fixed with formaldehyde following ultrasonic fragmentation. Next, STAT3 antibody, positive control Pol RNA II antibody, or negative control IgG antibody were added to the fragmented mixtures, respectively. The region containing the STAT3 binding site was amplified by PCR with specific primers. (**B**) HEK293 or LS174T cells were co-transfected with pGL3−1826 and pRL-TK, and different concentrations of AG490 were added into the culture medium 1 h before hTFF3 addition. The relative fluorescence intensity was detected after 24 h. Data are presented mean ± S.D. (*P < 0.05 compared with the sample treated with 0 μM AG490).

In order to confirm the interaction between STAT3 and the hTFF3 promoter, ChIP assay was performed. After fixation, sonication, immunoprecipitation, reversal of link, PCR, and other steps, PCR products were subjected to DNA gel electrophoresis. Our results showed that intense DNA bands were detected in the STAT3 ChIP sample and the input sample, while no DNA band was detected in the negative control, IgG ChIP (Fig. 4A). AG490 is a specific inhibitor of the transcription factor STAT3, which can specifically block the transcription activity of STAT3 protein^5^,^6^. Luciferase activity assay demonstrated that AG490 could potently inhibit the self-induction of hTFF3 on its own promoter (Fig. 4B).

### Transcriptional activation of the hTFF3 promoter by STAT3

**Figure 5:**
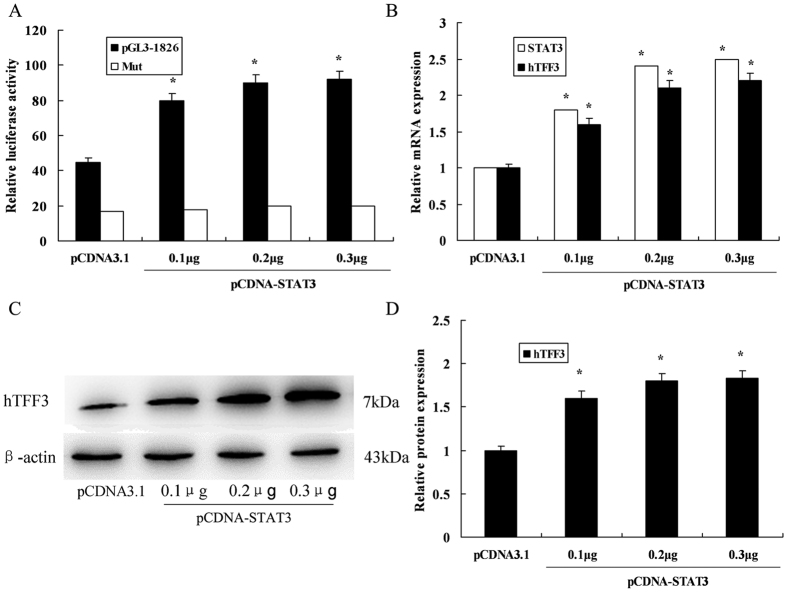
The effect of STAT3 overexpression on hTFF3. pGL3−1826 or mutant pGL3−1826 and STAT3 overexpression plasmids were co-transfected into LS174T cells, and hTFF3 was added at 24 h post-transfection, followed by continuous culture for 24 h. (**A**) Detection of relative fluorescence intensity. (**B**) Real-time analysis of STAT3 and hTFF3 mRNA levels. (**C,D**) Western blot analysis of hTFF3 protein levels. Data are presented mean ± S.D. (*P < 0.05 compared with the sample expressing pCDNA3.1).

**Figure 6:**
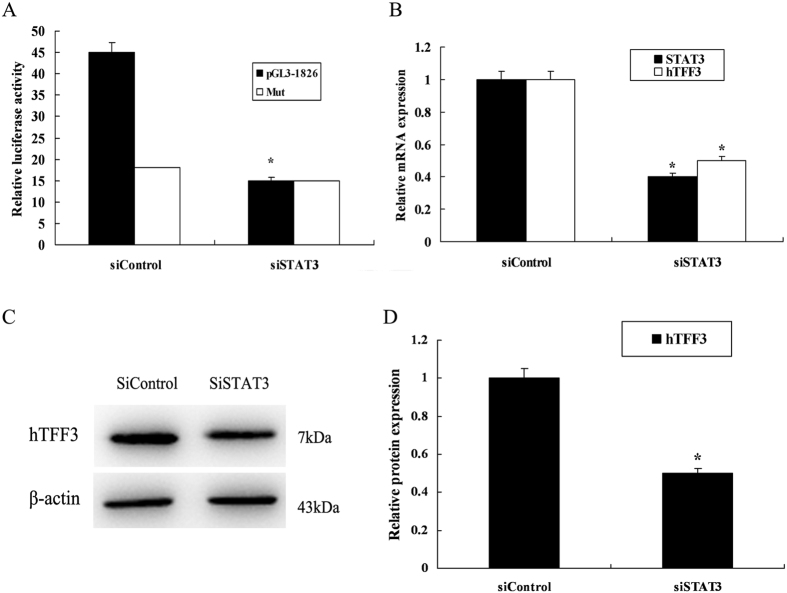
The effect of STAT3 knockdown on hTFF3. pGL3−1826 or mutant pGL3−1826, and STAT3 siRNA were co-transfected into LS174T cells, and hTFF3 were added at 24 h post-transfection, followed by continuous culture for 24 h. (**A**) Detection of relative fluorescence intensity. (**B**) Real-time analysis of STAT3 and hTFF3 mRNA levels. (**C,D**) Western blot analysis of hTFF3 protein levels. Data are presented mean ± S.D. (*P < 0.05 compared with the sample expressing pCDNA3.1).

To determine whether the transcription of hTFF3 promoter was affected by STAT3 upregulation, pGL3−1826 or mutant pGL3−1826 and STAT3 eukaryotic expression vectors were co-transfected. We found that luciferase activity of cells expressing the hTFF3 promoter gradually increased with increasing amounts of STAT3 plasmid in a dose-dependent manner. While the luciferase activities of cells expressing mutant pGL3−1826 did not show the increase (Fig. 5A). Real-time RT-PCR analysis showed that mRNA expression of hTFF3 was significantly elevated upon STAT3 overexpression (Fig. 5B). Western blot results also demonstrated upregulation of hTFF3 at the protein level (Fig. 5C,D). Conversely, RNAi knockdown was used to downregulate STAT3 expression, and the promoter activity and expression of hTFF3 were determined. The results showed that transcription of the hTFF3 promoter significantly decreased upon STAT3 RNAi knockdown. While co-transfection of mutant pGL3−1826 with STAT3 knockdown plasmids did not decrease the luciferase activities (Fig. 6A). Real-time RT-PCR analysis showed that both STAT3 and hTFF3 mRNA levels were decreased in the STAT3 knockdown cells (Fig. 6B). Western blot results also showed that protein expression of hTFF3 was significantly decreased upon STAT3 RNAi knockdown (Fig. 6C,D).

## Conclusions

Intestinal mucosal injury underlies the pathogenesis of many diseases^7^. Therefore, it is of great importance to maintain the integrity of the intestinal mucosa. hTFF3 is a small polypeptide of potential therapeutic value, and its main pharmacological action is to ameliorate gastrointestinal mucosal injuries caused by various factors and to promote repair of the damaged mucosa^8^,^9^,^10^. There are six cysteine residues in the amino acid sequence of hTFF3, connected in pairs by disulfide bonds to form three ring structures. This structure makes it stable, thus resistant to acidic and basic conditions, and protease hydrolysis. The structural stability protects hTFF3 from damages in the gastrointestinal tract, which is a complex environment, and also enables physiological functions of hTFF3. A variety of substances has been found to regulate hTFF3 expression by the corresponding response elements, such as, upstream stimulatory factor (USF), interleukin, and hypoxia inducible factor 1 (HIF-1)^11^,^12^,^13^,^14^. However, its basic regulatory mechanisms remained unclear. This study validated the self-induction effect of hTFF3 on its own promoter, and identified the functional region for the action of self-induction. We also identified the transcription factors binding to the hTFF3 promoter, which will help us elucidate the regulatory mechanisms of hTFF3 expression. This study confirmed the transcriptionally enhancing effect of hTFF3 on its promoter. The results showed that with increasing amounts of hTFF3, the transcription of the hTFF3 promoter was gradually enhanced in a dose-dependent manner, indicating that hTFF3 has a strong self-induction effect on its own promoter. When hTFF3 concentration was more than 50 μg/mL, transcription of the hTFF3 promoter entered into a plateau stage, thus the concentration of hTFF3 used in the subsequent experiments was 50 μg/mL (Fig. 1). To search for the functional region of the hTFF3 promoter important for its self-induction, truncated fragments of the hTFF3 promoter were tested by luciferase assay. Our results demonstrated that the transcriptional activities in cells expressing promoter fragments of −1826 bp and −1519 bp were restively high; while the activities of cells expressing fragments downstream of −1070 bp decreased significantly, suggesting that the functional region of hTFF3 was located between −1519 bp to −1070 bp. To identify the core region of the hTFF3 promoter, eight different truncated vectors between −1519 bp and −1070 bp were constructed. The results showed that cells expressing the −1450 bp promoter fragment exhibited higher activity, and cells expressing the promoter fragment with the region of −1400 bp to −1100 bp exhibited significantly decreased activity. The results show that the core functional region of the hTFF3 promoter could be narrowed down to −1450 bp to −1400 bp. Subsequently, we performed bioinformatics analysis of the hTFF3 promoter and searched for transcription factors in the TFSEARCH database. The search revealed that a STAT3 binding site (TTCCTGGAA) was discovered in the −1417 bp to −1409 bp region, with a score of 94.2. Therefore, the core region of the STAT3 binding site was mutated (CTG to ACT). As shown in Fig. 3, the luciferase activity of cells expressing pGL3−1826 was over 45-fold higher than that of cells expressing pGL3-basic, while the activity of cells expressing the mutant reporter decreased to only 17-fold of cells that express pGL3-basic. Therefore, we speculate that −1417 bp to −1409 bp is the core region of hTFF3 for its self-induction mechanisms. STAT3 is a shuttle protein, which is present in the cytoplasm in the absence of stimulus, and can translocate into the nucleus to bind specific DNA sequences upon activation^15^. STAT3 has dual functions in signal transduction and transcriptional regulation^16^. The STAT3 protein is widely expressed in different types of human tissues and cells, and is involved in cell proliferation, differentiation, apoptosis, and a variety of physiological functions^17^. It is also associated with the physiological and pathological functions of inflammation, tumor, and immune response^18^,^19^,^20^. To test whether the self-induction effect of hTFF3 was affected by the STAT3 binding site at −1417 bp to −1409 bp, we first performed ChIP on the hTFF3 promoter (pGL3−1826), which is the best way to study the binding activity *in vivo* between transcription factors and promoters. The results showed that STAT3 can bind to the hTFF3 promoter *in vivo*. Next, we used a specific STAT3 inhibitor, AG490, to block the binding activity of STAT3 to the hTFF3 promoter. Results of the luciferase reporter assay showed that AG490 could significantly reverse the self-induction effect of hTFF3 on its promoter activity in cells treated with different concentrations of AG490 for 24 h. All of the above results confirmed that STAT3 can bind to the hTFF3 promoter. Subsequently, the regulatory effect of STAT3 on hTFF3 self-induction was determined by STAT3 overexpression and knockdown assays. A eukaryotic expression vector of STAT3 was constructed and co-transfected with pGL3−1826 into LS174 cells. We found that transcription of the hTFF3 promoter and expression levels of hTFF3 were enhanced by co-transfection of STAT3 in a dose-dependent manner. In contrast, when STAT3 expression was decreased by RNAi knockdown, we found that the transcription of the hTFF3 promoter decreased by 40% in STAT3 knockdown cells. Real time RT-PCR analysis showed that the STAT3 mRNA expression level decreased to approximately 40% of the level before knockdown and that the hTFF3 mRNA levels were downregulated to approximately 50% of the level before knockdown. Western blot analysis showed that upon RNAi knockdown, hTFF3 protein expression also decreased significantly. In summary, this study first confirmed the self-induction effect of hTFF3. By amplifying the hTFF3 promoter to generate truncated mutants of varying lengths, the functional region (−1450 bp to −1400 bp) of the hTFF3 promoter was determined. Bioinformatics analysis confirmed a STAT3 binding site located in the region of −1417 bp to −1409 bp, and site-directed mutagenesis analysis revealed that this binding site was essential for the self-induction effect of hTFF3. ChIP experiments proved that in the presence of hTFF3, STAT3 binds to the hTFF3 promoter. Furthermore, STAT3 overexpression and knockdown assays demonstrated that STAT3 enhanced the self-induction effect of hTFF3 on its own promoter and the expression of hTFF3.

## Materials and Methods

### Cell culture

Human embryonic kidney (HEK) cell line HEK293 and colon cancer cell line LS174T were purchased from ATCC (Manassas, VA, USA). Cells were cultured in DMEM supplemented with 10% fetal bovine serum, and penicillin and streptomycin (100 U/mL) at 37 °C in 5% CO2. Culture medium was replaced every other day and cells were passaged every 3–4 days at a ratio of 1:3. Properly shaped cells were used for experiments.

#### Generation of the full-length hTFF3 promoter construct and truncated mutant constructs

Considering the full-length of the hTFF3 promoter (−1826 to +19 bp) as a template, primers targeting the 5′ -untranslated region (5′ -UTR) sequence (AB038162) of hTFF3 in GenBank were designed, and KpnI and HindIII enzyme cutting sites were introduced in the upstream and downstream primers, respectively. hTFF3 promoter fragments of different lengths (truncation mutants) were amplified and subsequently subjected to double enzyme digestion with KpnI and HindIII. The purified DNA fragments were ligated with pGL3-basic vector, and positive clones were selected for enzyme digestion with KpnI and HindIII. Finally, these constructs were subjected to sequence confirmation. AliBaba 2.1 software was used to analyze the transcription factor binding sites to ensure that novel binding sites were not introduced into the constructs.

### Construction of STAT3 expression vector

Total RNA extracted from HEK293 cells was reverse-transcribed into cDNA. The STAT3 gene sequence (NM_139276) was obtained from GenBank, and Primer 5.0 software was used to design the following primers: forward, 5′-CCCAAGCTTATGGCCCAATGGAATCAGCT-3′; and reverse, 5′-CCGCTCGAGTCACATGGGGGAGGTAGCGC-3′. PfuUltra™ DNA polymerase (Stratagene, USA) was used to amplify the STAT3 gene. PCR products were double-digested with HindIII and XhoI, and cloned into the pCDNA3.1(+) vector. The ligated clones were subjected to double digestion and sequencing.

### Double-stranded small interfering RNA (siRNA)

RNA interference was used to silence endogenous STAT3 expression. Control siRNA and human STAT3-specific siRNA were purchased from Santa Cruz (Santa Cruz, CA, USA), and the experiments were conducted according to the manufacturer’s instructions.

### Cell transfection and luciferase assay

HEK293 and LS174T cells undergoing logarithmic growth were seeded in 96-well plates, and were subjected to transfection with jetPEI reagent kit (Polyplus-Transfection, France) at 80% confluence. The ratio of jetPEI to plasmid DNA was 2:1. Control pGL3, pGL3-hTFF3, and pGL3 basic plasmids were transfected at 100 ng/well. Transfection of each plasmid was performed in triplicate wells, and 3 ng of pRL-TK plasmid was co-transfected in each well as a loading control. Transfection medium was replaced with fresh medium six hours after transfection. At 48 h post-transfection, different concentrations of hTFF3 protein were added, and cells were collected for analysis after 48 h. AG490, a small molecule inhibitor of STAT3-DNA binding, was added to the medium prior to hTFF3 treatment. In STAT3 overexpression and knockdown experiments, 100 ng luciferase reporter plasmid, 3 ng pRL-TK plasmid, and STAT3 overexpression or knockdown plasmids were transfected into cells, and different concentrations of hTFF3 were added into the culture medium at 24 h-post transfection. Cells were collected for analysis at 48 h post-transfection. Luciferase activity was measured using the dual reporter assay system (Promega, Madison, Wisconsin, USA), and relative fluorescence intensity was defined as the ratio of the firefly fluorescence intensity to *Renilla* fluorescence intensity. Three independent experiments were performed for each experimental condition.

### Chromatin immunoprecipitation (ChIP) Assay

ChIP assays were performed using the ChIP-IT kit (Active Motif, USA) according to the manufacturer’s instructions. HEK293 cells or LS174T cells were routinely cultured to 70–80% confluence, and subsequently fixed by medium containing 1% formaldehyde. After rinsing with pre-chilled PBS, glycine was added to the cells to stop fixation, the cells were rinsed again with pre-chilled PBS. Next, the fixed cells were collected for cell lysis followed by centrifugation to collect the cell nuclei. The centrifuged nuclei were re-suspended and sonicated by ultrasound to shear the chromatin into ~500 bp fragments. After treatment with RNase A and proteinase K, the effect of chromatin shearing was determined by agarose gel electrophoresis on a 1% agarose gel. Protein G beads were used to clear unspecific antibody binding in the chromatin lipid, and STAT3 antibody was subsequently added to the cleared chromatin. RNA Pol II and IgG antibodies were used as positive and negative controls, respectively. The mixture was incubated at 4 °C overnight, followed by the addition of Protein G beads. After washing, the formaldehyde crosslinks in the eluted antibody-chromatin complexes were reversed, and DNA was purified. The purified DNA was then amplified by PCR. The PCR primers were: forward, 5′-CAGAGGCTCCTGGAAGGG-3′; and reverse, 5′-CAACCTCCTGCAGTGGAC-3′ (143 bp product).

### Quantitative real-time RT-PCR

Total RNA was extracted from cells using TRIzol reagent (Invitrogen, New York, CA, USA) according to the manufacturer’s instructions. Total RNA was reverse-transcribed into cDNA using the PrimeScript RT Master Mix (Perfect Real Time) Kit (TaKaRa, Dalian, China). Using cDNA as a template, two pairs of primers were used for real-time PCR analysis. hTFF3 primers were forward, 5′-CCAAGGACAGGGTGGACTG-3′; and reverse, 5′-AAGGTGCATTCTGCTTCCTG-3′. STAT3 primers were forward, 5′-ATCACGCCTTCTACAGACTGC-3′; and reverse, 5′-CATCCTGGAGATTCTCTACCACT-3′. PCR reaction system with 20 μL volume: 0.5 μL cDNA template, 0.25 μL forward primer, 0.25 μL reverse primer, 10 μL RNase-free ddH2O, 8 μL 2.5 × Real Master Mix (SYBR Green I). Reaction conditions: pre-denaturation: 95 °C for 10 s, 1 cycle; PCR reaction: 95 °C for 15 s, 60 °C for 60 s, 40 cycles. Statistical analysis was performed following data collection.

### Western blot

Cells were collected and lysed, and total protein was quantified using BCA Protein Quantification Kit (Pierce, USA). Forty micrograms of total protein was loaded in each lane, and a 15% SDS-PAGE gel was run to separate the proteins. After electrophoresis, proteins were transferred onto a cellulose nitrate membrane, and incubated with anti-hTFF3 or anti-STAT3 polyclonal antibodies, or anti-β-actin monoclonal antibody (Abcam, Cambridge, MA, USA), respectively, overnight at 4 °C. Subsequently, the membrane was incubated with horseradish peroxidase-labeled anti-mouse IgG secondary antibody at room temperature for 2 h. Chemiluminescence signals were quantified using an ECL imager, and analyzed using Quantity One software (Bio-Rad, Hercules, CA, USA).

### Statistical Analysis

Statistical analysis was performed using SPSS Software (version 16.0). Data are presented as mean ± S.D. Results were analyzed using unpaired *t* tests, and P < 0.05 was considered significant.

## Additional Information

**How to cite this article**: Sun, Y. *et al*. Human Trefoil Factor 3 induces the transcription of its own promoter through STAT3. *Sci. Rep.* **6**, 30421; doi: 10.1038/srep30421 (2016).
